# Community pharmacists’ perceptions on multidisciplinary heart failure care: an exploratory qualitative study

**DOI:** 10.1186/s12913-023-09661-8

**Published:** 2023-06-14

**Authors:** Willem Raat, Pauline Truyts, Justine Gaillaert, Marie Van de Putte, Lorenz Van der Linden, Stefan Janssens, Bert Vaes, Miek Smeets

**Affiliations:** 1 Department of Public Health and Primary Care, KU Leuven, Kapucijnenvoer 7, blok D bus 7001 3000, Leuven, Belgium; 2 Department of Pharmaceutical and Pharmacological Sciences, KU Leuven, Leuven, Belgium; 3 Zorgzaam Leuven, Leuven, Belgium; 4 Pharmacy Department, University Hospitals Leuven, KU Leuven, Leuven, Belgium; 5 Department of Cardiovascular Diseases, University Hospitals Leuven, KU Leuven, Leuven, Belgium

**Keywords:** Heart failure, Pharmacologist, Multidisciplinary care, Qualitative design

## Abstract

**Background:**

Heart failure (HF) is an important health problem and guidelines recommend multidisciplinary management. The pharmacist is an important member of the multidisciplinary heart failure team, both in the hospital and community setting. This study aims to explore the perceptions of community pharmacists on their role in HF care.

**Methods:**

We conducted a qualitative study based on face-to-face semi-structured interviews with 13 Belgian community pharmacists between September 2020 and December 2020. We used the Qualitative Analysis Guide of Leuven (QUAGOL) method as guidance for data analysis until data saturation was reached. We structured interview content into a thematic matrix.

**Results:**

We identified two major themes: heart failure management and multidisciplinary management. Pharmacists feel responsible for the pharmacological and non-pharmacological management of heart failure, citing easy access and pharmacological expertise as important assets. Diagnostic uncertainty, lack of knowledge and time, disease complexity and difficulties in communication with patients and informal care providers are barriers to optimal management. General practitioners are the most important partners in multidisciplinary community heart failure management, although pharmacists perceive a lack of appreciation and cooperation and deplore communication difficulties. They feel intrinsically motivated to provide extended pharmaceutical care in HF but cite the lack of financial viability and information sharing structures as important barriers.

**Conclusion:**

The importance of pharmacist involvement in multidisciplinary heart failure teams is undisputed by Belgian pharmacists, who cite easy access and pharmacological expertise as important assets. They point out several barriers impeding evidence-based pharmacist care for outpatients with heart failure: diagnostic uncertainty and disease complexity, lack of multidisciplinary information technology and insufficient resources. We recommend that future policy should focus on improved medical data exchanges between primary and secondary care electronic health records as well as the reinforcement of interprofessional relationships between locally affiliated pharmacists and general practitioners.

**Supplementary Information:**

The online version contains supplementary material available at 10.1186/s12913-023-09661-8.

## Introduction

Heart failure (HF) is an important and global health concern due to the rising prevalence and an increase in risk factors and comorbidities in an ageing patient population [1]. Consequently, multidisciplinary care programs, which focus on the management of multimorbidity and chronicity, are at the core of recent research efforts [2]. Such programs are recommended with the highest degree of evidence (class IA in the most recent guideline of the European Society of Cardiology) [3]. An important member of the multidisciplinary heart failure team is the pharmacist, both in the hospital and in the ambulatory setting. Inclusion of a pharmacist has been shown to reduce risk in HF and all-cause hospitalizations and is recommended by the American College of Cardiology [4–6]. The pharmacist’s role in the care of patients with HF could include medication reconciliation and education; medication initiation; dosage titration, adjustment, and monitoring; participation in disease management pathways and follow-up after hospital discharge [7]. The implementation of these aspects of care is regionally varied, the provision of pharmaceutical care in community pharmacy is still limited within Europe and has an emphasis on routine general activities such as patient screening [8].

Several reviews and meta-analyses have indicated that incorporation of pharmacists’ care results in better medication adherence, disease knowledge and improvement of some measures of quality of life in outpatients with HF [6, 9–12]. However, the success of implementing community pharmacist care for HF patients in outpatient settings is largely dependent on how these health care providers perceive their own role in the multidisciplinary care framework. For example, a pharmacist might not wish to engage in multidisciplinary care due to fear of care fragmentation [13]. Other determinants for successful uptake of pharmacist care in this domain are adequate training, sufficient staffing, supportive national legislation, and local/regional partnerships with other health care providers [14]. In the national setting of our study, Belgium, for example, there have been several initiatives in the last decade to strengthen the role of community pharmacists, in part to allow the provision of care closer to each citizen. First, there is special remuneration for pharmaceutical services for patients with asthma (i.e., pharmaceutical consultations) and for the appointment of a ‘home pharmacist’ (since October 2017). A major task of home pharmacists is to regularly update and document the patient’s medication regimen, while rendering it accessible to other care providers [15]. The home pharmacist’s remuneration is contingent on a signed informed consent form by the patient. Secondly, multidisciplinary cooperation between doctors and pharmacists is also encouraged through the reimbursement of medico-pharmaceutical consultations (i.e., MFOs).

There has been a wealth of qualitative design studies on the views of both physicians and pharmacists on the role of community pharmacists in delivering clinical services [16–19], noting the need for incentives and better communication to improve multidisciplinary care as well as complex stakeholder relationships. However, no such research has been conducted on the way community pharmacists perceive their role in routine HF care. This study aims to explore these perceptions.

## Methods

### Design

We used an exploratory qualitative design to study pharmacists’ perceptions. We took a phenomenological approach [20] and conducted face-to-face semi-structured interviews. The consolidated criteria for reporting qualitative research (COREQ) checklist was used to report our findings [21].

### Participants and recruitment

Fifteen community pharmacists were asked to participate. We aimed to balance gender, setting (urban, rural), and years of experience (purposive sampling). With regards to purposive sampling, it is important to note that almost 80% of all community pharmacists in Flanders are female [22] and that of the 22 boroughs constituting Leuven, only one has a rural character [23]. One researcher (MVP) had pre-existing access to data on gender and years of experience on practicing community pharmacists in the Leuven municipality through her role as coordinator of an integrated chronic care program in this region [24]. This data helped in selecting pharmacists following our sampling frame and extending invitations to take part in this study. We initiated contact by phone and email. One month after the initial contact, we sent a reminder via email. In the absence of a response, we contacted pharmacists in person to ask for voluntary participation in the study. No remuneration was provided.

### Setting

The study was conducted in the municipality of Leuven, Belgium, an urbanized region of approximately 100 000 inhabitants with a federally supported integrated care pilot program called “Zorgzaam Leuven” [24]. This program implements vicinity networks of health care professionals and mixed financing models to improve chronic care. The program also embedded a complex disease management intervention for HF [25]. Belgium has a dense network of community pharmacists (1 per 2253 persons in 2019), staffed by at least one pharmacist. Pharmacists perform or supervise a multitude of pharmaceutical acts, including advice on the correct use of medication, and providing the necessary information in relation to health promotion and disease prevention. Pharmacists also refer to other health care providers upon indication [15].

### Data collection

Our methods for data collection and analysis were similar to those described in Raat et al. [26]. Two of the authors (PT and JG) conducted face-to-face semi-structured interviews between September 2020 and December 2020. One of the authors functioned as interviewer, the other as observer taking notes. We chose this approach to maximize the familiarity of researchers with each interview and context. Both researchers were female and were completing their final year of pharmacy training at the time of the interviews. They were not familiar with the interviewees within a professional context, although interviewees were aware that they were pharmacy students. MS trained both authors in conducting interviews. The interviews took place in the participating pharmacists’ pharmacies. We used a topic list that was based on the literature [2, 9, 26, 27] and discussions between the authors to structure the interviews (supplemental files: Topic list). Five authors were involved in the development of the topic list (WR, MS, MVP, PT, JG). Two had experience in the development of a topic list for interview studies (WR, MS) and a third (MS) was involved in the coordination of the local integrated care program. The interview with the very first participant (lasting approx. 45 min) was used to pilot and refine the topic list further. The data from this interview were also included in the analysis. All interviews were conducted in Dutch, the native language of the interviewers and pharmacists, and all audio was recorded. The analysis was conducted using the original transcripts in Dutch and a professional language editor without knowledge of the topic translated the selected illustrative quotes from the interviews for publication. We conducted interviews until we reached data saturation. We defined data saturation as the moment upon which two previous interviews no longer contributed any new elements and when a certain category had been exhaustively described in all its dimensions and variations [28].

### Data analysis

All interviews were transcribed verbatim, including both verbal and non-verbal signs (conversation analysis) [29]. We adhered to the principles of the Qualitative Analysis Guide of Leuven (QUAGOL) as guidance in data analysis [30]. The procedure consisted of two steps: (1) a preparatory phase using word processing software and (2) the actual coding process, facilitated by NVIVO 12 software (QSR International, Melbourne Australia).

The aim of the preparatory phase was to become as familiar as possible with the interview data in order to compile a list of concepts as starting point for the coding in NVIVO 12. First, two authors (PT and JG) captured the context and essence of each interview separately based on the interview transcript and unedited notes and then discussed this together. Afterwards, a conceptual scheme was drafted for each interview. The usability of this scheme was monitored by repeated comparisons with the interviews. Finally, the research team (WR, PT, JG, MS) analyzed and compared the conceptual schemes of the various interviews to compile a list of concepts.

Two authors (PT and JG) performed the coding process using NVIVO 12 software. First, they coded the data by linking each fragment of text to one of the concepts in the list. Consequently, PT and JG generated an initial coding tree in the program. The relevance and usefulness of the codes and concepts were evaluated by the research team (WR, PT, JG, MS) and adjusted when necessary. In the final step, three researchers (PT, JG and MS) separately extracted the essential storyline from the data. After reaching consensus, the findings were discussed within the entire research team.

We used investigator triangulation in a group of diverse researchers across all stages of the analytical process to increase the reliability of our results.

### Reflexivity

The research team consisted of three GPs (WR, MS, BV), two pharmacists in training (PT, JG), a community pharmacist (MVdP), a hospital pharmacist (LVdL), and a cardiologist (SJ). The impetus for this research came from the observation in two researchers’ PhD projects (WR, MS) [25, 31, 32] that community pharmacists were rarely involved in multidisciplinary disease management. Since both researchers are GPs, we wanted to include pharmacists in the research since they might have a different viewpoint and counteract ours. We therefore engaged two researchers in the final year of pharmacy training to conduct the interviews as part of their formal education master thesis. In addition, we recruited a community pharmacist, a hospital pharmacist, and a cardiologist to offer written feedback on the manuscript. WR, MS, BV, MVP, LVL and SJ have all been involved in several projects investigating multidisciplinary HF care and integrated chronic care more generally, both in hospital and community settings. They have a shared passion for multidisciplinary HF care. Extensive oral and written discussion throughout the project between the involved researchers led them to reflect on their own clinical practice as well as to a consensus on the structure and contents of the discussion. Although most of the research team holds research positions, WR, MS, BV, LVL and SJ are primarily engaged in patient care, and we think this increased the validity of our interpretations.

### Ethical considerations

We obtained approval from the ethical committee of the University Hospital Leuven (MP015495) on August 28th, 2020. All pharmacists received an information letter and gave written informed consent.

## Results

Thirteen community pharmacists participated in the study. Their characteristics are listed in Table 1. Data saturation was reached after the thirteenth interview. There was a variety in years of experience and practice sizes. However, participants were predominantly female (n = 12) and active in an urban setting (n = 12). The average interview length was 50 min.

**Table 1:** Characteristics of participating pharmacists

Pharmacist	Sex	Years of experience	Pharmacy size (active full-time equivalents)	Setting
1	Female	30	1	Urban
2	Female	15	2	Urban
3	Female	23	1.5	Urban
4	Female	15	1.8	Urban
5	Female	8	1	Urban
6	Female	33	2	Urban
7	Female	17	2	Urban
8	Female	21	2	Urban
9	Female	2	3	Urban
10	Male	14	2	Rural
11	Female	19	3	Urban
12	Female	26	5	Urban
13	Female	12	7	Urban, located next to hospital

We structured interview content into a thematic matrix (Table 2). We identified two major themes and three minor themes that pertained to each major theme. The first major theme was the management of heart failure by the pharmacist. This first theme refers to all facets of heart failure management the pharmacist dealt with in his or her pharmacy, without necessarily involving other health care practitioners. Our second major theme was multidisciplinary heart failure management. This second theme refers to all facets of heart failure management that pertained to the cooperation with other health care professionals. Within these major themes, we identified three minor themes. The first one was pharmacists’ ‘current role’, meaning how they deal with the management of heart failure currently. The second one was ‘barriers to care provision’, meaning barriers to the provision of care by the pharmacist to the patient. The third one was ‘future perspectives’, meaning how pharmacists perceive their future role in HF management.

**Table 2:** Thematic matrix

Theme	Pharmacist heart failure management	Multidisciplinary heart failure management
Current role	Pharmacological management Non-pharmacological management Low-threshold access	GP as the most important partner Modes of communication
Barriers to care provision	Diagnostic uncertainty Lack of knowledge and time Communication with patients and informal care providers Disease complexity	Lack of appreciation Communication difficulties Lack of cooperation
Future perspectives	Increased role for the home pharmacist Increased time	Information sharing Information platforms

### Theme 1 – Pharmacist heart failure management

#### Role of the pharmacist

Participants expressed an important role in the pharmacological management of heart failure. This entailed drafting medication schedules, logging medication records and interactions, as well as informing patients of medication importance and possible side effects.

*“We look at what medications they should take, what dose and we prepare a schedule for them. If they return from the physician, we check if and what has changed. In fact, we are confidants and very accessible. Sometimes it is easier for them to tell us things than to a physician or other care providers.” (Pharmacist 6)*

Most participants also articulated a responsibility in the non-pharmacological management of heart failure. This meant detecting signs and symptoms of heart failure worsening as well as providing lifestyle advice (i.e., salt restriction).

*“If you say, look, I am going to give you some tips that can help, then usually it is possible to address such things with the patient.” (Pharmacist 8)*

*Participating pharmacists considered themselves as very accessible to patients, thus lowering the barrier to care in the HF care pathway and improving detection of symptoms that would signal decompensation or medication issues. “We see the patient much more often. You frequently hear from the patient: ‘Yes, I did not dare to ask the GP, because he is looking at his watch and there is a new patient every ten minutes’. But here they abide!” (Pharmacist 4)*

#### Barriers to care provision

A major pharmacist-related barrier to care was uncertainty regarding the diagnosis of heart failure. One respondent noted that many patients find this to be a frightening term. Most respondents had difficulty assessing possible heart failure based purely on medication combinations and noted that this was exacerbated by problems in communication and information sharing with the GP, which were recurring themes as well.

*“What I do find difficult about heart failure is, sometimes we don’t know who has heart failure. Because I find it hard to ask: ‘Do you have heart failure?’ You can have some suspicion based on the medication, but if they are not open about it, I find it difficult to talk about. I am also unsure whether physicians always tell their patients whether they have heart failure or not. I sometimes don’t know whether the patient is cognizant of the term, so they are able to correspond about it. Also, because we are not offered access to physicians’ medical records, so we must notice it ourselves as care providers” (Pharmacist 9)*

Many respondents noted difficulties in the management of heart failure because of a lack of disease-specific knowledge.

*“Heart failure, yes, it is not so easy. First you need to acquire and sustain proper learning. I think that this is lacking among pharmacists of my age and older. When I see how you (interviewers) are trained now, we never had that.” (Pharmacist 12)*

Respondents noted several important patient-related barriers to providing heart failure care. Patients were sometimes very reluctant to share health information. Pharmacists encountered difficulties when navigating between those patients who treated them as mere commercial drug dispensers and those who saw them as responsible for their entire medication plan.

*“Patients are sometimes so closed and reserved that care is very difficult. You perform a full medication review and the result is a complete shutdown of the patient.” (Pharmacist 3)*

Since many heart failure patients suffer from reduced mobility, their medication is often picked up from the pharmacy by informal care providers. This led to frustrations due to the inability to communicate directly with the patient.

*“Say you whisper something in someone’s ear, and they have to convey this information… By the time you reach the final person half the information will be lost or false. We can try, but it remains difficult.” (Pharmacist 13)*

Another important barrier was the complexity of heart failure medication schedules and the efforts needed to keep them up to date.

*“Two years ago, my intern made a master thesis on keeping medication schedules up to date. But that is a difficult one in practice. There should be a pop-up, so you cannot advance before updating the schedule in case of new medications.” (Pharmacist 4)*

Equally important for optimal provision of care was the perceived lack of time.

*“The biggest influencing factor is how many patients are waiting in the pharmacy. We should be receiving more time or space for this, but in everyday practice this is not always feasible.” (Pharmacist 7)*

The registration of pharmaceutical consultations was administratively cumbersome.

*“I’m having a hard time with the fact that we must publicize this officially. You must get it signed; you must book an appointment. People do not always want this. Asking: ‘Do you want to sign here? Then I am going to give you the education.’ It does not make sense!” (Pharmacist 7)*

#### Future perspectives

Most pharmacists stressed the importance of increasing investment (time and funding) in their role as a home pharmacist. Since current funding is almost exclusively based on sale volumes, there is a clear financial conflict when pharmacists invest time to provide chronic care consultations. Of course, this means an uneasy equilibrium between the role of health care professional and commercial agent, and in this light the previous comments regarding patients’ reluctance to share health information are perhaps not as surprising. Pharmacists want to engage in chronic care services but are disincentivized to do so.

*“In the ideal world you reserve half a day each week to do a consultation every half hour on appointment. You must have time and knowledgeable personnel. Also, space, a tablet computer, a brochure... I think this can also be performed by a specialized staff member” (Pharmacist 12)*

*“I think this should be reimbursed. Because we perform many services without reimbursement. We must sell a box and then we receive our money. If we want to perform this role, it must be adequately reimbursed, otherwise the business model is unsustainable.” (Pharmacist 13)*

This also entails the need for materials that facilitate tailored delivery of care.

*“In the manual it literally says: look here you have this or that. Come on, it is made for toddlers! I find it hard to say to the patient: ‘Now I’m going to explain what is going on with you.’ We need a booklet on which we as pharmacists can base ourselves to conduct such a consultation, not a guidebook you have to place between yourself and the patient.” (Pharmacist 2)*

### Theme 2 – Multidisciplinary heart failure management

#### Role of the pharmacist

Respondents noted that the most important partner in the multidisciplinary management of heart failure was the GP. Consequently, this was the most important partner for multidisciplinary communication, which consisted of requests in case of problems or questions. This understandably led to a feeling of unidirectional communication rather than collaboration.

*“I find this very important, and I hope this will happen more and more often in the future. What I experience here in the pharmacy, and for which I joined Zorgzaam Leuven, is that the physicians of whom I see the most patients, are not open to this. Or rather, they are open to it but unwilling to make time for it.” (Pharmacist 7)*

Many pharmacists stressed the importance for GPs to acknowledge their expertise in the patients’ interest. As mentioned in the previous theme, not only is there no financial incentive to provide HF care, but there is also no multidisciplinary agreement or understanding to engage community pharmacists in a HF pathway.

*“But physicians also have to stimulate their patients to visit the community pharmacy and ask for information. Patients confide more in their physician than in a pharmacist. If that physician provides more information to the patient, they will be more open to the idea” (Pharmacist 10)*

*“I think that physicians still not grasp what pharmacists could be doing. That’s why these projects are useful, but everyone still remains on their own islands while they would be better off cooperating.” (Pharmacist 11)*

Contact with community nurses or other health care providers was limited, exacerbating the feeling of exclusion from a multidisciplinary team.

*“(On non-physician care providers) We don’t communicate with them at all.” (Pharmacist 2)*

*“There should be a way for us to know which nurses are coming there. Often, they are rotating nurses. We could ask the physician but bothering them for such a thing...” (Pharmacist 10)*

*“I’m in contact with a few home nurses, well, actually for one or two patients mostly. They call occasionally. Er, as for the rest, less really. They are not always very reachable.” (Pharmacist 5)*

Pharmacists used different modes of communication to contact GPs or other health care providers. Most preferred contact by telephone. Privacy-secured instant messaging apps, while deemed sufficient to receive prescriptions, were not considered to be useful in daily communication and even less so in a team environment.

*“It is not easy communicating extensively with a doctor. That is why I prefer Siilo.(a secure instant messaging app, red.) You can answer when you find the time and, more extensively as well” (Pharmacist 10)*

*“I find the app very easy. You don’t have to call or disturb the doctor when it is not urgent. It is secured, conforms to privacy laws and the physicians can respond when they have time” (Pharmacist 1)*

*“I receive messages on my personal smartphone device, my team does not have access when I’m off work.” (Pharmacist 3)*

Most pharmacists questioned the use of the medico-pharmaceutical consultation (MFO) due to lack of follow-up.

*“I connected well with several nurses on a night like that, but then it stops. Currently, this is very difficult, but I hope this will change” (Pharmacist 7)*

*“I find it difficult because the message you are giving them, is this transmitted or not?” (Pharmacist 1)*

#### Barriers to care provision

Many pharmacists felt a lack of appreciation for their role as health care providers. This perceived lack of appreciation stems from several of the previously identified themes.Specifically, the lack of reimbursement or structural support to provide HF care and the lack of means to communicate with other health care disciplines or access to relevant health information.

*“I have the sense you are not being appreciated for the work you are doing” (Pharmacist 6)*

*“I think our role in primary care is of supreme importance, but it is undervalued.” (Pharmacist 3)*

*“It’s been two years that we’re addressing that there should be an extra place for the home pharmacist in nurses’ patient diary, just as there is one for a physician. So they know directly what’s up and so you don’t have to repeat everything ten times.” (Pharmacist 8)*

The most important barriers to adequate multidisciplinary management of heart failure were the difficulties in contacting treating physicians and the lack of adequate communication instruments to share health information or medication schedules.

Many pharmacists commented on the difficulty reaching physicians (during in-office hours), as well as a hesitancy to call out of fear of disturbing a consultation.

*“I think a physician could be a bit more approachable. Now you just have a telephone number, and you know for sure that when you are calling them that they are in consultation and that you are bothering them tremendously. Whereas it is not an urgent question, but there is no other way to pose it... That is totally different compared with pharmacists. They often have a phone number, e-mail address, even a personal phone number with WhatsApp. Many more possibilities. It is annoying the lengths which one must go to in order to contact a physician” (Pharmacist 13)*

This problem was less pronounced in group practices and some respondents noted an improvement in younger practices.

*“Reaching the physician, it’s not always possible. But these days they return a call. They would never have done that in the past. Sometimes they pass you their personal phone number. I feel I can’t complain because it has improved a lot lately. I do feel that physicians are trying their best for that.” (Pharmacist 8)*

Although pharmacists felt themselves to be crucial in the transition of care after discharge, communication with physicians in the hospital or GP on-call service was perceived to be almost impossible.

*“Communication with the hospital is even more important, but they are unreachable. Even the GP out-of-hour medical post is unreachable to us. Meaning, we can’t call directly to the physicians sitting there. You reach the front desk where all callers end up and then you have to explain in which capacity and why you are calling.” (Pharmacist 2)*

Pharmacists commented on the dismal performance of the current eHealth infrastructure (Vitalink) to share and update medication schedules between health care providers.

*“Something that is only half functioning actually makes things harder, because if you can’t trust the system, it is useless.” (Pharmacist 3)*

This frustration extended to the lack of communication regarding heart failure diagnoses and relevant health information.

*“Yes, in principle it is the GP who should communicate this to us, but I get… Hm, at the moment there just isn’t a channel, other than via the patient, where it is easy to do so, I think.” (Pharmacist 5)*

*“I think we can do more if we are allowed to know more… For example, being allowed to know what is going on… If you don’t know what is happening it is hard to do anything about it.” (Pharmacist 3)*

#### Future perspectives

*Information sharing*

Most pharmacists were adamant on the potential of increased access to relevant medical information to improve health care outcomes. Particularly in the transitional care setting where patients were often discharged with altered chronic medication schedules.

*“Often patients with heart failure are admitted to the hospital, or if the GP can intervene early it is not necessary, but often it is. If they would just make an informational package for the pharmacist… That would be a good first step for us to get more involved.” (Pharmacist 9)*

*“I still miss that we know very little about it (hospital admissions).” (Pharmacist 7)*

For even the most basic of information elements such as conveying the presence of HF or the rationale behind changes in dosages of chronic medication compounds, pharmacists proposed communicating via prescription in the absence of more structural information technology platforms.

*“But I am never certain if the doctor does not add it in writing. Actually, I think that, as a physician, you should write it down on the prescription. Just ‘HF’, not ‘heart failure’. The patient does not know what it means, but at least we do.” (Pharmacist 1)*

*“We sometimes have to believe what the patient is saying. We don’t have glycemic values or anything. We see prescriptions but we don’t have a full picture of the medication schedule. That makes it hard. Then you call two or three times to the physician… That could be improved upon. If there are major changes, they communicate it to us by letter, or on a prescription with information. This extends to all conditions of course.” (Pharmacist 9)*

*Information platforms*

Pharmacists were unanimous regarding the role of an improved information infrastructure as a way of transcending current communication barriers. They envisioned a shared and convenient pharmaceutical electronic health record that integrated both pharmaceutical and medical care elements. The most important elements would be relevant diagnoses, lab values such as renal clearance and brief physician reports.

*“The collaboration between care providers and health care itself could be made more efficient if more is shared through software. I think mostly about software that allows to share information, proper communication, renal clearance, lab values, etc.…” (Pharmacist 1)*

*“It should be possible to find out the most important disease of a patient via a single button after reading a prescription. With discharge medication it is often very difficult to guess what is going on, because often patients don’t know themselves. Sometimes you have to guess at the indication or disease.” (Pharmacist 12)*

## Discussion

The aim of this study was to investigate how community pharmacists perceive their role in routine HF care. Our findings indicate that they feel they could play an important role in the provision of multidisciplinary heart failure care, citing easy access and pharmacological expertise as important assets. They point out several barriers impeding evidence-based pharmacist care for outpatients with heart failure: diagnostic uncertainty and disease complexity, lack of multidisciplinary information technology and insufficient resources.

To our knowledge, this is the first study to investigate the views of community pharmacists on multidisciplinary routine care for heart failure specifically. Our findings are in line with the conclusions of several other studies investigating community pharmacists’ views on multidisciplinary care in general which sampled a broad range of Western primary care settings and qualitative methods. A first major conclusion in this paper is that community pharmacists want an active role in HF management. This relates to a previous study where pharmacists tend to position themselves as bridge-builders and problem-solvers, actively engaged in patient care and a last line of defense in the prevention of medication errors [33]. This proactive view is corroborated by a recent meta-analysis on pharmacists’ care in outpatients with heart failure, which found consistent improvements in medication adherence and knowledge, physical function and disease and medication management [9]. Evidence-based pharmacist outpatient care includes medication review, adherence support, supporting symptom control and patient education on HF management [9]. This paper shows that the implementation of these services in our context was non-existent. A second major conclusion of this study is that participating community pharmacists do not feel that they are supported to engage in active HF management. As was reported in several studies, they expressed a need for additional training and resources [17, 34], as well as access to patient health information (e.g. medical records) [34] as important points for improvement and policy planning.

However, additional funding for extended pharmacy practices will likely prove contentious if the underlying differences in perspective on multidisciplinary collaboration between physicians (mostly general practitioners) and pharmacists persist. Community physicians are inclined to see pharmacists as a useful checkpoint, but in a passive health care role as non-clinicians [33], and are liable to compete for services such as flu vaccination or to be suspicious of pharmacists’ financial motives [17]. This is perhaps unsurprising since the current economic model for community pharmacists (in Belgium) is almost entirely based on sales volume and thus unlikely to incentivize the provision of pharmaceutical care. This model means that the amount of time a community pharmacist must interact with patients at the counter renders disease management or therapeutic management non-viable [35]. Such a financial structure is prevalent in Europe, where less than half of all countries reimburse pharmaceutical services [36], and countries that implement and maintain pharmaceutical services within a viable business model such as the UK have significantly improved provision of pharmaceutical care [8]. An example of a tangible benefit are minor ailment schemes in the UK, which are cost-effective and reduce the burden of minor ailments on high-cost settings such as general practice and emergency care. In addition to the lack of financially incentivized cooperation, interprofessional relationships are weak and there are few natural meeting arenas or collaboration opportunities, other than occasional phone calls [33].

We offer two possible avenues for concrete policy implementation. First, interprofessional education (IPE) has been proposed as a possible solution for improved multidisciplinary understanding, but our study suggests that its current implementation does little to unite patients’ own health care providers. The current program could benefit from a greater participation of complex chronic patients’ own home pharmacists and particularly their general practitioners, who constitute a key member of the HF team along with the specialist HF nurse [37]. This could be incentivized through stipends or accreditation credits and integrate joint medication reviews, as already implemented in the UK [38]. Implementation of this form of collaboration showed reduced medication burden and drug related problems as well as significantly improved interprofessional communication in a community setting [39–41], as well as in nursing homes [42]. Interestingly, although pharmacists think this type of applied interdisciplinary collaboration should be stimulated where possible, they feel it should not be obligatory since this might cause them to exclude patients from some GPs from this service (due to lack of interest in working with GPs they dislike) [43]. Second, pharmacists should be given access to relevant medical data, particularly on current medication and relevant medical conditions (such as HF). Such an approach has been shown to improve patient outcomes [44–47]. Our results suggest that the current medication data exchanges frustrate pharmacists and need an overhaul. In addition, there should be an effort to integrate the exchange of relevant data within the existing electronic health records of pharmacists, general practitioners, and hospitals. There already exists a template for the contents of such an exchange [48]. Pending this complex and larger effort, general practitioners should be sensibilized to communicate a heart failure diagnosis as a free text field into the already existing and mandatory Belgian electronic Recip-e prescribing system [49].

### Strengths and limitations

To our knowledge, this is the first qualitative study to investigate the views of community pharmacists on heart failure specifically. We used the robust QUAGOL method to guide data analysis through continuous discussions among the participating authors, capturing the essence of each interview before starting the actual coding process. There are some limitations to this study. First, we used purposive sampling in a region which is urban, and which already has a high level of multidisciplinary health care provider engagement through a federally sponsored pilot program. However, this should presumably bias our results positively, whereas we encountered the same obstacles as previous research. Second, participants were predominantly female and based in urban settings (only one male and one rural community pharmacist agreed to participate).We believe that the imbalance in gender was reasonable since this corresponds with the national distribution of community pharmacists (more than 7 out of 10 are women [22, 50]). The same goes for the imbalance in setting, since the region in which we recruited community pharmacists has only one borough out of twenty-two with a rural character [23]. This proves the challenge in identifying what constitutes rural or urban in a densely populated region such as central Flanders (820 inhabitants/m2 in the urban region with 4 million inhabitants between Gent, Brussels, Antwerp and Leuven). For example, only 9 boroughs transcended the population density criterion (5000 inhabitants/m²) used to define urban settings in Belgium and none reached the limit used in the United Kingdom (10.000) or the Netherlands (20.000)[51]. Third, the two interviewers were novices in qualitative research and interview techniques. This was compensated for by extensive training in semi-structured interview techniques and qualitative data analysis, as well as the use of investigator triangulation. Fourth, we conducted the interview sessions during an ongoing coronavirus pandemic that significantly strained the community health care system and impacted continuity of care. This may have negatively impacted pharmacists’ perceptions on their role in multidisciplinary care. It may also have made recruitment more difficult due to the strain imposed on primary care health systems. Finally, there was little discussion of the role of cardiologists and other secondary or tertiary health personnel, although the transitional care setting is especially important for pharmacists considering medication changes after HF hospitalizations or outpatient cardiology visits. This does, however, illustrate the current lack of communication and collaboration noted by pharmacists.

## Conclusions

The importance of pharmacist involvement in multidisciplinary heart failure teams is undisputed by Belgian pharmacists, who cite easy access and pharmacological expertise as important assets. They point out several barriers impeding evidence-based pharmacist care for outpatients with heart failure: diagnostic uncertainty and disease complexity, lack of multidisciplinary information technology and insufficient resources. We recommend that future policy should focus on improved medical data exchanges between primary and secondary care electronic health records as well as the reinforcement of interprofessional relationships between locally affiliated pharmacists and general practitioners.

## Electronic supplementary material

Below is the link to the electronic supplementary material.

Supplementary Material 1

## Data Availability

The datasets used and/or analyzed during the current study are available from the corresponding author on reasonable request.

## References

[1] Groenewegen A, Rutten FH, Mosterd A, Hoes AW (2020). Epidemiology of heart failure. Eur J Heart Fail.

[2] Takeda A, Martin N, Taylor RS, Taylor SJ (2019). Disease management interventions for heart failure. Cochrane Database Syst Rev.

[3] McDonagh TA, Metra M, Adamo M, Gardner RS, Baumbach A, Böhm M (2021). 2021 ESC Guidelines for the diagnosis and treatment of acute and chronic heart failure: developed by the Task Force for the diagnosis and treatment of acute and chronic heart failure of the European Society of Cardiology (ESC) with the special contribution of the heart failure Association (HFA) of the ESC. Eur Heart J.

[4] Milfred-Laforest SK, Chow SL, Didomenico RJ, Dracup K, Ensor CR, Gattis-Stough W (2013). Clinical pharmacy services in heart failure: an opinion paper from the heart failure society of America and American College of Clinical Pharmacy Cardiology Practice and Research Network. J Card Fail.

[5] Dunn SP, Birtcher KK, Beavers CJ, Baker WL, Brouse SD, Page RL 2, et al. The role of the clinical pharmacist in the care of patients with cardiovascular disease. J Am Coll Cardiol. 2015;66(19):2129–39. 10.1016/j.jacc.2015.09.025 26541925

[6] Koshman SL, Charrois TL, Simpson SH, McAlister FA, Tsuyuki RT (2008). Pharmacist Care of Patients with Heart failure: a systematic review of Randomized trials. Arch Intern Med.

[7] Cheng JWM, Cooke-Ariel H (2014). Pharmacists’ role in the care of patients with heart failure: review and future evolution. J Managed Care Pharm.

[8] Hughes CM, Hawwa AF, Scullin C, Anderson C, Bernsten CB, Björnsdóttir I (2010). Provision of pharmaceutical care by community pharmacists: a comparison across Europe. Pharm World Sci.

[9] Schumacher PM, Becker N, Tsuyuki RT, Griese-Mammen N, Koshman SL, McDonald MA (2021). The evidence for pharmacist care in outpatients with heart failure: a systematic review and meta-analysis. ESC Heart Failure.

[10] Cao VFS, Cowley E, Koshman SL, MacGillivray J, Sidsworth M, Turgeon RD. Pharmacist-led optimization of heart failure medications: A systematic review. JACCP: JOURNAL OF THE AMERICAN COLLEGE OF CLINICAL PHARMACY. 2021;4(7):862 – 70.

[11] Parajuli DR, Kourbelis C, Franzon J, Newman P, McKinnon RA, Shakib S (2019). Effectiveness of the pharmacist-involved Multidisciplinary Management of Heart failure to Improve Hospitalizations and Mortality Rates in 4630 patients: a systematic review and Meta-analysis of Randomized controlled trials. J Card Fail.

[12] Schulz M, Griese-Mammen N, Anker SD, Koehler F, Ihle P, Ruckes C (2019). Pharmacy‐based interdisciplinary intervention for patients with chronic heart failure: results of the PHARM‐CHF randomized controlled trial. Eur J Heart Fail.

[13] Lowrie R, Johansson L, Forsyth P, Bryce SL, McKellar S, Fitzgerald N (2014). Experiences of a community pharmacy service to support adherence and self-management in chronic heart failure. Int J Clin Pharm.

[14] van Mil JWF, de Boer WO, Tromp THFJ (2011). European barriers to the implementation of pharmaceutical care. Int J Pharm Pract.

[15] Gerkens S, Merkur S, Belgium (2020). Health Syst Rev Health Syst Transit.

[16] Bryant LJM, Coster G, Gamble GD, McCormick RN (2009). General practitioners’ and pharmacists’ perceptions of the role of community pharmacists in delivering clinical services. Res Social Administrative Pharm.

[17] Hindi AMK, Jacobs S, Schafheutle EI (2019). Solidarity or dissonance? A systematic review of pharmacist and GP views on community pharmacy services in the UK. Health Soc Care Commun.

[18] Hurley E, Gleeson LL, Byrne S, Walsh E, Foley T, Dalton K (2021). General practitioners’ views of pharmacist services in general practice: a qualitative evidence synthesis. Fam Pract.

[19] Löffler C, Koudmani C, Böhmer F, Paschka SD, Höck J, Drewelow E (2017). Perceptions of interprofessional collaboration of general practitioners and community pharmacists - a qualitative study. BMC Health Serv Res.

[20] Savin-Baden M, Major CH (2012). Chapter 14 - phenomenology. Qualitative research: the essential guide to theory and practice.

[21] Tong A, Sainsbury P, Craig J (2007). Consolidated criteria for reporting qualitative research (COREQ): a 32-item checklist for interviews and focus groups. Int J Qual Health Care.

[22] RIZIV. Apothekers op de arbeidsmarkt. 2019.

[23] Leuven. Wijk in Cijfers [Available from: https://leuven.incijfers.be/dashboard/dashboard/wijk-in-cijfers/.

[24] Van de Putte M, Van Pottelbergh G (2022). Towards integrated care in the complex healthcare system of Belgium: an implementation strategy based on vicinity networks and a mixed financing model. Int J Integr Care.

[25] Raat W, Smeets M, Van Pottelbergh G, Van de Putte M, Janssens S, Vaes B. Implementing standards of care for heart failure patients in general practice - the IMPACT-B study protocol. Acta Cardiol. 2020;Accepted for publication. 10.1080/00015385.2020.1844504 33161831

[26] Raat W, Smeets M, Vandewal I, Broekx L, Peters S, Janssens S (2021). Cardiologists’ perceptions on multidisciplinary collaboration in heart failure care-a qualitative study. BMC Health Serv Res.

[27] Smeets M, Zervas S, Leben H, Vermandere M, Janssens S, Mullens W (2019). General practitioners’ perceptions about their role in current and future heart failure care: an exploratory qualitative study. BMC Health Serv Res.

[28] Bernard HR, Wutich A, Ryan GW (2017). Analyzing qualitative data: systematic approaches.

[29] Mortelmans D. Handboek kwalitatieve onderzoeksmethoden. Acco; 2013.

[30] Dierckx de Casterle B, Gastmans C, Bryon E, Denier Y (2012). QUAGOL: a guide for qualitative data analysis. Int J Nurs Stud.

[31] Smeets M, Vaes B, Aertgeerts B, Raat W, Penders J, Vercammen J et al. Impact of an extended audit on identifying heart failure patients in general practice: baseline results of the OSCAR-HF pilot study. ESC Heart Fail. 2020. 10.1002/ehf2.12990 PMC7754725 32969599

[32] Raat W, Smeets M, Janssens S, Vaes B (2021). Impact of primary care involvement and setting on multidisciplinary heart failure management: a systematic review and meta-analysis. ESC Heart Fail.

[33] Rakvaag H, SØreide GE, Meland E, Kjome RL (2020). Complementing or conflicting? How pharmacists and physicians position the community pharmacist. Pharm Pract (Granada).

[34] Hindi AMK, Schafheutle EI, Jacobs S (2019). Community pharmacy integration within the primary care pathway for people with long-term conditions: a focus group study of patients’, pharmacists’ and GPs’ experiences and expectations. BMC Fam Pract.

[35] Gregório J, Cavaco AM, Lapão LV (2017). How to best manage time interaction with patients? Community pharmacist workload and service provision analysis. Res Social Administrative Pharm.

[36] Martins SF, Van Mil J, Da Costa FA (2015). The organizational framework of community pharmacies in Europe. Int J Clin Pharm.

[37] Morton G, Masters J, Cowburn PJ (2018). Multidisciplinary team approach to heart failure management. Heart.

[38] Barnes E, Ashraf I, Din A (2017). New roles for clinical pharmacists in general practice. Prescriber.

[39] Lelubre M, Wuyts J, Maesschalck J, Duquet N, Foubert K, Hutsebaut C (2019). Implementation study of an intermediate medication review in belgian community pharmacies. Res Social Administrative Pharm.

[40] Wuyts J, Maesschalck J, De Wulf I, De Lepeleire J, Foulon V (2020). Studying the impact of a medication use evaluation by the community pharmacist (Simenon): patient-reported outcome measures. Res Social Administrative Pharm.

[41] Wuyts J, Maesschalck J, De Wulf I, Lelubre M, Foubert K, De Vriese C (2020). Studying the impact of a medication use evaluation by the community pharmacist (Simenon): drug-related problems and associated variables. Res Social Administrative Pharm.

[42] Strauven G, Anrys P, Vandael E, Henrard S, De Lepeleire J, Spinewine A (2019). Cluster-controlled trial of an intervention to improve prescribing in nursing homes study. J Am Med Dir Assoc.

[43] Wuyts J. Exploration of pharmaceutical care services to support adequate medication use in older polymedicated patients in primary care and post-discharge. 2019.

[44] Lankford C, Dura J, Tran A, Lam SW, Naelitz B, Willner M (2021). Effect of clinical pharmacist interventions on cost in an integrated health system specialty pharmacy. J Managed Care Specialty Pharm.

[45] Nelson SD, Poikonen J, Reese T, El Halta D, Weir C (2017). The pharmacist and the EHR. J Am Med Inform Assoc.

[46] Gernant SA, Zillich AJ, Snyder ME (2018). Access to medical records’ impact on community pharmacist–delivered medication therapy management: a pilot from the medication safety research network of indiana (Rx-SafeNet). J Pharm Pract.

[47] Snyder JM, Ahmed-Sarwar N, Gardiner C, Burke ES (2020). Community pharmacist collaboration with a primary care clinic to improve diabetes care. J Am Pharmacists Association.

[48] Wuyts J, Ginste MV, De Lepeleire J, Foulon V (2020). Discharge report for the community pharmacist: development and validation of a prototype. Res Social Administrative Pharm.

[49] Nyssen M, Putzeys T, Baert L, Buckens M, editors. Recip-e: the electronic prescription system for ambulatory care in Belgium2014; Cham: Springer International Publishing.

[50] Caerels V. Zeven op de tien apothekers zijn vrouwen De Apotheker: Roularta; 2019 [Available from: https://www.deapotheker.be/actualiteit/zeven-op-de-tien-apothekers-zijn-vrouwen/article-normal-37005.html.

[51] How is an urban area defined?. [Available from: https://ourworldindata.org/urbanization.

