# Supplementary material for: Community pharmacists’ perceptions on multidisciplinary heart failure care: an exploratory qualitative study
Source: BMC Health Serv Res. 2023 Jun 14;23:638. doi: 10.1186/s12913-023-09661-8 (PMC10266313; doi:10.1186/s12913-023-09661-8)
Supplement: Supplementary file 1 — Supplementary Material 1 [file 12913_2023_9661_MOESM1_ESM.docx]

Supplemental files

Topic list
How would you describe your role as health care professional in the care for patients with heart failure?

1. In general
   1. How do you find it to recognize patients with heart failure?
      1. What works well? What makes it harder?
   2. How do you find it to be a home pharmacist?
      1. How well do you achieve this role?
   3. What do you think of the role you currently play in patients with heart failure?
      1. What could go better according to you? What do you suggest to better take up this role as a health care professional?
2. More specifically with regards to pharmacological management
   1. How does the delivery of medication go with you? What do you do regarding medication during delivery of a drug? What is your experience with pharmacist patient consultations?
   2. What do you think of the role you are currently performing for heart failure patients with regards to pharmacological management?
      1. What could be improved, according to you? What do you suggest facilitating taking up this role as health care provider?
3. More specifically with regards to non-pharmacological management
   1. What questions or messages will you deliver to the patient?
      1. To what do you pay attention when you know you are dealing with a heart failure patient?
      2. What could be improved, according to you? What do you suggest to facilitate taking up this role as health care provider?

What influencing factors do you experience in taking up this expanded role in the care of patients with heart failure?

1. How does … influence taking up this role as health care provider?
   1. Caregiver
   2. Knowledge
      1. According to you, what can be done to optimize this?
   3. Lack of time
      1. According to you, what can be done to optimize this?
      2. How would you reorganize your work to make time?
   4. Location requirements
   5. Reimbursement

Would you recall your last heart failure patient, please? What influenced that episode?

How do you see your current role in the multidisciplinary treatment of patients with heart failure?

- What does that multidisciplinary collaboration currently entail? Is there multidisciplinary collaboration and with which health care professionals?
- What is your vision on the importance of multidisciplinary collaboration with patients with a chronic illness such as heart failure?
- What are your current experiences with multidisciplinary collaboration? Do physicians, care coordinators and teams contact you?
- What are your current experiences with interprofessional education sessions (MFO’s)?
- What are factors impeding this multidisciplinary cooperation?
- What is your vision on medication data exchanges such as Vitalink?

How do you see the pharmacists’ role evolving in the future?
Towards the patient?
With regards to multidisciplinary collaboration?
What extra requirements do you think are necessary to facilitate this collaboration?
How do you see this evolve?
